# Fueling genome maintenance: On the versatile roles of NAD^+^ in preserving DNA integrity

**DOI:** 10.1016/j.jbc.2022.102037

**Published:** 2022-05-17

**Authors:** Joanna A. Ruszkiewicz, Alexander Bürkle, Aswin Mangerich

**Affiliations:** Molecular Toxicology Group, Department of Biology, University of Konstanz, Konstanz, Germany

**Keywords:** NAD, DNA repair, PARPs, ARTs, sirtuins, ADPR, ADP-ribose, ART, ADP-ribosyl transferase, ARTD, diphtheria toxin–like ADP-ribosyltransferase, ATM, ataxia–telangiectasia mutated, BER, base excision repair, CD, cluster of differentiation, CS, Cockayne syndrome, DSB, double-strand break, LIG, ligase, NA, nicotinic acid, NAAD, nicotinic acid adenine dinucleotide, NADK, NAD^+^ kinase, NAM, nicotinamide, NAMPT, nicotinamide phosphoribosyl transferase, NAR, nicotinic acid riboside, NHEJ, nonhomologous end-joining, NMN, nicotinamide mononucleotide, NMNAT, nicotinamide mononucleotide adenylyl transferase, NR, nicotinamide riboside, PAR, poly-ADP-ribose, PARP, poly-ADP-ribose polymerase, PARylation, poly(ADP-ribosyl)ation, ROS, reactive oxygen species, SIRT, sirtuin, Trp, tryptophan, XPA, xeroderma pigmentosum group A

## Abstract

NAD^+^ is a versatile biomolecule acting as a master regulator and substrate in various cellular processes, including redox regulation, metabolism, and various signaling pathways. In this article, we concisely and critically review the role of NAD^+^ in mechanisms promoting genome maintenance. Numerous NAD^+^-dependent reactions are involved in the preservation of genome stability, the cellular DNA damage response, and other pathways regulating nucleic acid metabolism, such as gene expression and cell proliferation pathways. Of note, NAD^+^ serves as a substrate to ADP-ribosyltransferases, sirtuins, and potentially also eukaryotic DNA ligases, all of which regulate various aspects of DNA integrity, damage repair, and gene expression. Finally, we critically analyze recent developments in the field as well as discuss challenges associated with therapeutic actions intended to raise NAD^+^ levels.

DNA is constantly exposed to a plethora of damaging factors of both exogenous and endogenous origin, such as replication stress, alkylating and oxidative molecules, or UV radiation. To protect DNA from lesions caused by such stressors and to ensure genomic stability, cells have developed several pathways that recognize and repair specific DNA lesions (Fig. 1). Thus, in particular, small lesions are subject to direct reversal mediated by single proteins, for example, O^6^-alkylguanine-DNA alkyltransferase repairs O-alkylated DNA damage, and the alkB homolog dioxygenases reverse N-alkylated base adducts (1). Yet, the majority of repair mechanisms involves multiple events mediated by different proteins and orchestrated in a complex network of DNA repair pathways: base excision repair (BER) targets small lesions like oxidized bases and apuric/apyrimidic sites, in a mechanism overlapping with single-strand break repair. Larger nucleotide adducts are removed by nucleotide excision repair, and mismatch repair targets errors produced during DNA replication and recombination. Double-strand breaks (DSBs) are repaired either through homologous recombination or nonhomologous end-joining (NHEJ) comprising a canonical pathway (C-NHEJ) or alternative end-joining (A-EJ). As a comprehensive review of DNA repair pathways falls outside the scope of this article, we refer the reader to other in-depth review articles on this topic (2, 3). The diversity of active repair mechanisms ensures DNA stability, but when it is impaired, the persistence of genomic errors may lead to severe cellular consequences, including senescence and cell death (2). In the long term, inefficient DNA repair and compromised genome maintenance contribute to carcinogenesis and determines the adaptational capacity of cancer cells as well as aging and age-related diseases (4, 5, 6).

**Figure 1:**
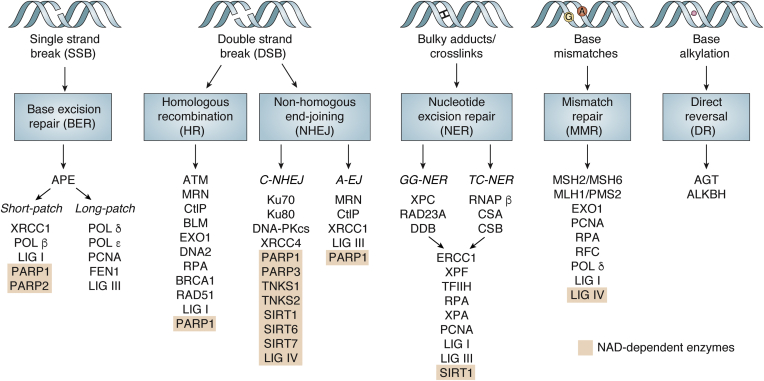
**Major DNA repair pathways.** DNA repair occurs *via* different pathways involving multiple proteins, among them many are directly NAD^+^ dependent (shaded in *orange*). Repair of single-strand breaks (SSBs) through base excision repair (BER) involves NAD^+^-consuming PARP1 and PARP2. Double-strand breaks (DSBs) are repaired either through homologous recombination (HR) or nonhomologous end-joining (NHEJ) pathways, facilitated by multiple ARTDs and SIRTs. NAD^+^-dependent enzymes are also implicated in nucleotide excision repair (NER) as well as mismatch repair (MMR) pathways. A potential NAD^+^-dependent role of LIG IV is under discussion (see text for details). ARTD, diphtheria toxin–like ADP-ribosyltransferase; LIG IV, ligase IV; PARP, poly-ADP-ribose polymerase; SIRT, sirtuin.

NAD^+^ fulfills multiple cellular roles, ranging from its function as a cofactor in energy production and redox regulation up to various signaling pathways essential for cell survival (7, 8). Interestingly, numerous NAD^+^-dependent reactions are involved in the preservation of genomic stability, cellular response to DNA damage, and other pathways concerning nucleic acids, such as gene expression or cell proliferation. Thus, the cellular NAD^+^ status influences genomic stability and sensitivity to DNA-damaging agents (9, 10, 11, 12, 13). Of note, NAD^+^ depletion in aging and age-related diseases implies supplementation of NAD^+^ boosting molecules as a plausible strategy in addressing age-dependent DNA damage (7, 14, 15).

Here, we first introduce the reader to the NAD^+^ biosynthesis and major cellular roles of this ubiquitous and versatile molecule. Furthermore, we turn our focus on the NAD^+^-dependent molecular mechanisms involved in mammalian genome maintenance. We concisely review both well-established concepts as well as new findings in the field. Finally, we discuss current controversies around NAD^+^-dependent therapeutic approaches that target various pathophysiological conditions in the light of its role in genome maintenance processes.

## NAD^+^ biosynthesis

**Figure 2:**
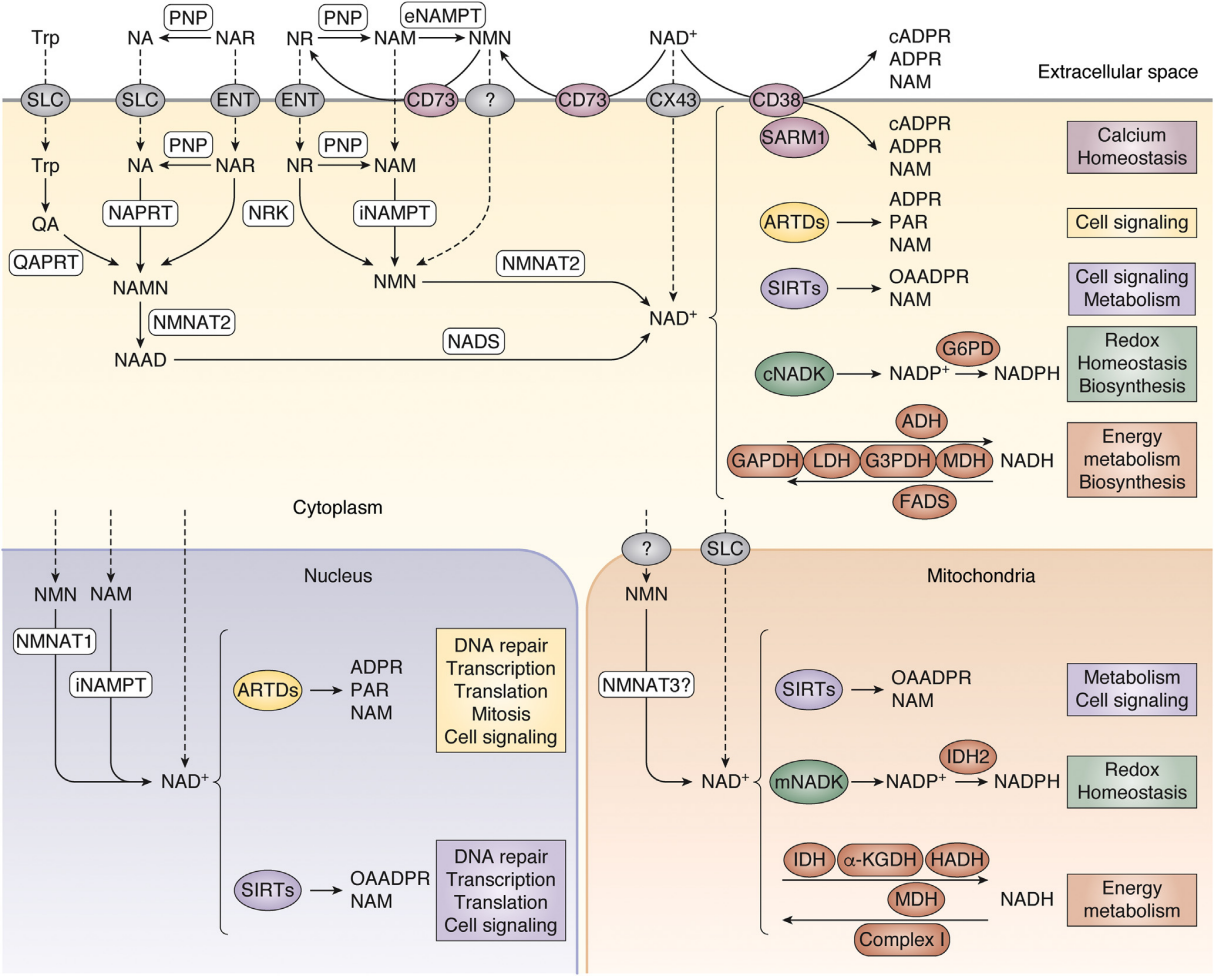
**NAD**^**+**^**biosynthesis and major cellular functions.** Cellular NAD^+^ levels are maintained by biosynthesis from dietary precursors: amino acid tryptophan (Trp) and vitamin B3, which comprises nicotinic acid (NA), nicotinamide (NAM), and nicotinamide riboside (NR). Trp is converted to quinolinic acid (QA) in the kynurenine pathway and further by quinolinate phosphoribosyltransferase (QAPRT) into nicotinamide mononucleotide (NAMN). NAMN is also produced from NA in the Preiss–Handler pathway by nicotinic acid phosphoribosyltransferase (NAPRT). Both pathways are completed by the transformation of NAMN into nicotinic acid adenine dinucleotide (NAAD) by nicotinamide mononucleotide adenylyltransferases (NMNAT1, NMNAT2, and NMNAT3) and further into NAD^+^ by NAD^+^ synthetase (NADS). The salvage pathway recycles NAM produced by NAD^+^-consuming enzymes: sirtuins (SIRTs), ADP-ribosyltransferases (ARTDs), as well NAD^+^ glycohydrolases and cyclic ADP-ribose synthases. NAM is transformed by nicotinamide phosphoribosyltransferase (NAMPT) into nicotinamide mononucleotide (NMN), which is then turned into NAD^+^*via* NMNATs. This pathway can be also fueled by NR, derived from diet or from dephosphorylation of nicotinamide mononucleotide (NMN). NAD^+^ levels are balanced by subcellular compartmentalization of NAD^+^ synthesis and consumption. In the cytoplasm and mitochondria, NAD^+^ is utilized *via* multiple pathways in bioenergetics, maintenance of redox homeostasis, or cell signaling. Whereas the nuclear NAD^+^ pool contributes in addition to maintenance of genomic homeostasis mainly *via* the NAD^+^-dependent ARTDs and SIRTs (see text for details).

In mammals, NAD^+^ is synthesized from various precursors ingested through the diet, such as forms of vitamin B_3_: nicotinic acid (NA), nicotinamide (NAM), and nicotinamide riboside (NR); as well as the essential amino acid tryptophan (Trp), which is metabolized *via* three major pathways: (i) salvage pathway, (ii) *de novo* pathway, and (iii) Preiss–Handler pathway (Fig. 2). In addition, alternative precursors such as nicotinic acid riboside (NAR) (16), biosynthesis intermediates, such as nicotinamide mononucleotide (NMN) (17), or products of NAD^+^ consumption (NAM, NMN, and NR) are salvaged to replenish cellular NAD^+^ pools. In order to supply cells with “fresh” exogenous precursors, those can enter the cell during a passive process (*e.g.*, NAM) or *via* membrane transporters—members of the solute carrier transporter family (for Trp and NA) or equilibrative nucleoside transporter (for NR and NAR). Furthermore, exogenous NMN can be taken up by cells, yet the cellular transport of this precursor molecule remains unclear (18, 19), with some studies suggesting that NMN uptake requires the prior conversion to NR by glycohydrolase, cluster of differentiation 73 (CD73) (20, 21).

The salvage pathway is a primary source of NAD^+^ in mammals (22) (Fig. 2). The first and rate-limiting step in this pathway—conversion of NAM to NMN, catalyzed by nicotinamide phosphoribosyl transferase (NAMPT)—is crucial in cellular NAD^+^ synthesis because numerous NAD^+^-consuming reactions release NAM. The enzyme NAMPT is present both extracellularly (eNAMPT) and intracellularly (iNAMPT) in the cytoplasm and nucleus, whereas its mitochondrial presence is disputed (23). Besides, NMN can be produced during ATP-dependent phosphorylation of NR by nicotinamide riboside kinase (17), and cleavage of the NR glycosidic bond by purine nucleoside phosphorylase produces NAM. Moreover, nicotinamide riboside kinase and purine nucleoside phosphorylase convert NAR to NAMN and NA, respectively, which feed the Preiss–Handler pathway (see later) (24). In the second (and final) step of the salvage pathway, NMN is converted to NAD^+^ by ATP-dependent nicotinamide mononucleotide adenylyl transferase (NMNAT). This enzyme in human tissues exists in three isoforms: the most ubiquitous nuclear NMNAT1, cytoplasmic NMNAT2 (25), and mitochondrial NMNAT3, whose function remains controversial (26, 27, 28). In addition, NMNAT catalyzes the formation of nicotinic acid adenine dinucleotide (NAAD) from NAMN, which comes from either Preiss–Handler or *de novo* pathways. In the Preiss–Handler pathway, NAMN is produced from NA by nicotinic acid phosphoribosyl transferase (29). In the *de novo* pathway, Trp is first transformed to quinolinic acid in the multistep process during the kynurenine pathway, and then quinolinic acid is converted to NAMN by quinolinic acid phosphoribosyl transferase. While originally thought to play a minor role in mammalian NAD^+^ synthesis, it has been recently suggested that the *de novo* pathway may play an important role in NAD^+^ homeostasis in humans (30, 31). In the final step of both Preiss–Handler or *de novo* pathways, NAAD is amidated to NAD^+^ by the cytosolic enzyme NAD^+^ synthase, which requires ATP, and ammonia or l-glutamine (32).

Cellular NAD^+^ levels are maintained in the range of 100 μM in the cytoplasm and nucleus and 200 μM in mitochondria (8). Such differences in subcellular NAD^+^ pools reflect its biological functions (see later) and are variable in tissues with different metabolic roles. Similarities in nuclear and cytoplasmic levels are likely because of the passive diffusion of NAD^+^ and its precursors through nuclear pores (33), although the detailed relationships between nuclear and cytoplasmic pools appear to be complex, since depletion of NAD^+^ in one compartment cannot be fully compensated by the other pool (28). As NAD^+^ has an overall negative charge, it is unable to passively cross lipid bilayers in mitochondria and other cellular membranes. Therefore, for a long time, the cytoplasmic/nuclear NAD^+^ pool has been thought to be strictly separated from the mitochondrial one, and it remained rather obscure how mitochondria build up their significant intraorganelle NAD^+^ levels. However, recently, this issue had been at least partially resolved. Thus, in 2020, a series of studies demonstrated the existence of a mitochondrial NAD^+^ carrier, that is, solute carrier transporter family SLC25A51 (or MCART1), providing mechanistic insight how mitochondria are supplied with NAD^+^ (28, 34, 35, 36).

The extracellular NAD^+^ presence, around 0.1 μM in mammals (37), is a result of either passive release from damaged cells (38) or active transport, for example, *via* connexin hemichannels like connexin 43 (39, 40). In turn, extracellular NAD^+^ can be degraded to precursors that can be taken up into the cell (41). Depending on the individual tissue, NAD^+^ molecules have a quite variable half-life that varies from 15 min to 15 h (42, 43, 44). Therefore, in tissues with high fluxes such as the small intestine and spleen, quick and efficient biosynthesis is necessary to compensate for a rapid cellular consumption *via* numerous NAD^+^-dependent reactions.

## Cellular roles of NAD^+^

Numerous biochemical reactions require NAD^+^ as a cosubstrate (Fig. 2). NAD^+^ and its reduced form (NADH) are critical regulators of cellular bioenergetics, serving as cofactors in reactions of glucose metabolism, fatty acid β-oxidation, or the tricarboxylic acid cycle. For example, NAD^+^ is reduced to NADH during glycolysis by GAPDH. In turn, NADH is oxidized by lactate dehydrogenase, when pyruvate is converted to lactate under anaerobic conditions. NADH is a cofactor for fatty acid desaturases, and NAD^+^ is essential for alcohol dehydrogenase. NAD^+^/NADH-dependent malate dehydrogenase (malate–aspartate shuttle) and glycerol-3-phosphate dehydrogenase (glycerol-3-phosphate shuttle) generate reducing equivalents transported across the membrane into the mitochondria. There, NAD^+^ is reduced by pyruvate dehydrogenase complex and the tricarboxylic acid cycle enzymes: malate dehydrogenase, α-ketoglutarate dehydrogenase, and isocitrate dehydrogenase. NAD^+^ is also reduced by hydroxyacyl-CoA dehydrogenase in β-oxidation during fatty acid metabolism. Subsequently generated NADH is the major reducing factor in complex I mitochondrial electron transport chain, for the transfer of electrons in oxidative phosphorylation that produces ATP. Thus, the NAD^+^/NADH abundance reflects the energy status of the cell (7, 45).

ATP-dependent NAD^+^ kinases (NADKs) in the cytosol (cNADK) and mitochondria (mNADK) phosphorylate NAD^+^ to NADP^+^, which together with its reduced form NADPH, is critical for the maintenance of cellular redox homeostasis. It regenerates molecules responsible for xenobiotic detoxification, for example, cytochrome P450, and eradication of reactive oxygen species (ROS), such as GSH, thioredoxin, or peroxiredoxin (46). NADP^+^ is also a cosubstrate for glucose-6-phosphate dehydrogenase, a key enzyme in the pentose phosphate pathway, which produces precursors for the synthesis of nucleotides and aromatic amino acids. Moreover, it contributes to the synthesis of fatty acids and nicotinic acid adenine dinucleotide phosphate, which serves as a second messenger for intracellular calcium (Ca^2+^) signaling (46, 47). Because of its reducing role, NADP^+^ is found predominantly in the reduced state, with NADP^+^/NADPH ratios 1:200, which contrasts the NAD^+^/NADH ratio of 1000:1 in the cytoplasm and of 10:1 in the mitochondria (48).

**Figure 3:**
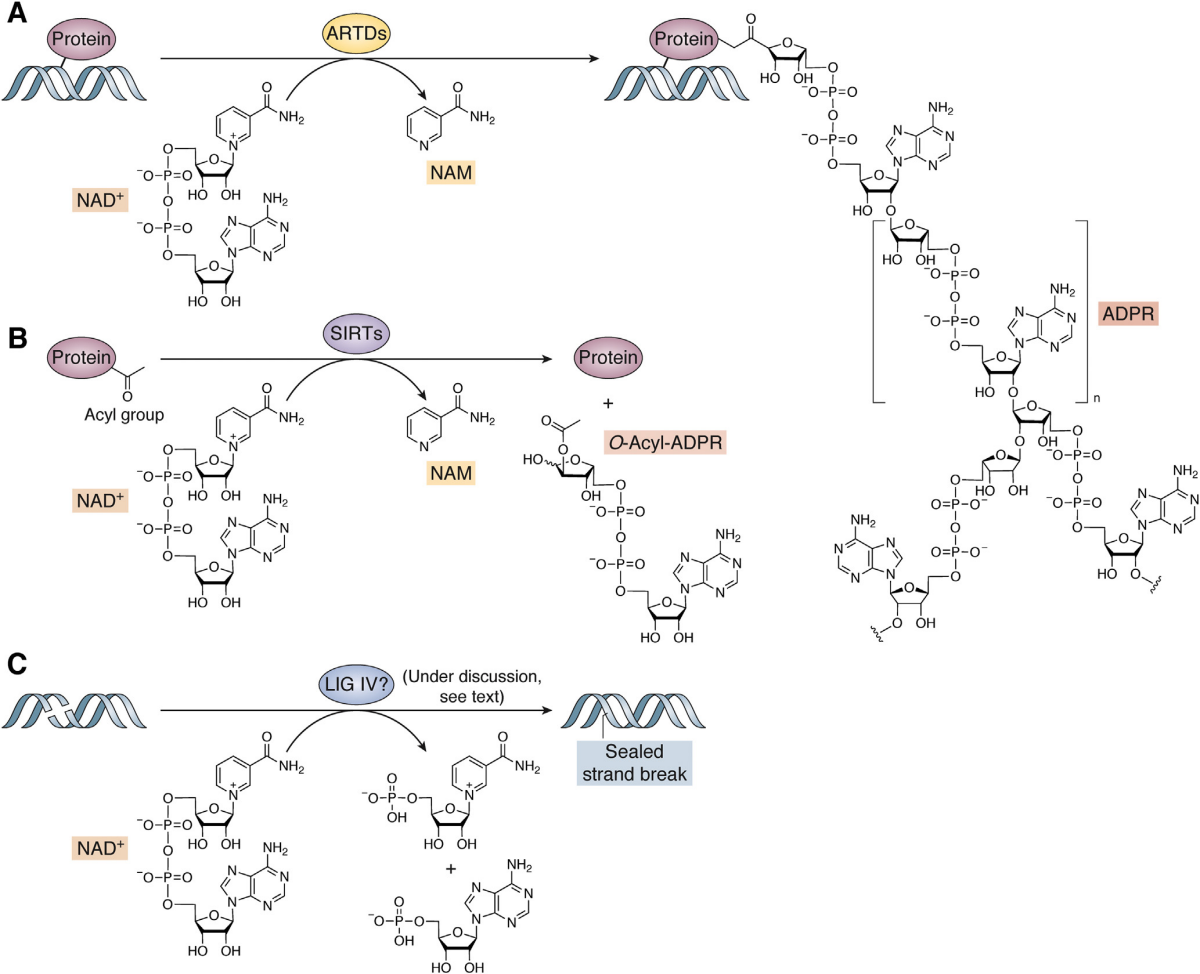
**NAD**^**+**^**-consuming reactions in genome maintenance.***A*, diphtheria toxin-like ADP-ribosyltransferases (ARTDs) catalyze the cleavage of N-glycosidic bond and covalently attaches ADP ribose (ADPR) moiety to target molecules: protein, DNA, or RNA. ARTD family members perform a transfer of either single ADPR units (MARylation) or multiple ADPR units (PARylation), connected in a linear or branched manner. *B*, sirtuins (SIRTs) mediate a transfer of an acyl group from protein to NAD^+^-derived ADPR, producing O-acyl-ADPR and NAM. *C*, DNA ligase IV (LIG IV) potentially uses α-phosphate moiety of NAD^+^ to form a new phosphodiester bond in the process of DNA nick repair. For references, see text.

In addition to the numerous reactions where NAD(P)^+^/NAD(P)H couples serve as an electron carrier, NAD^+^ can be also irreversibly consumed (Fig. 3). ADP-ribosyl transferases (ARTs) cleave the N-glycosidic bond, which releases NAM and ADP-ribose (ADPR) and further covalently attach the latter to the target molecule. Cholera toxin–like ADP-ribosyltransferases are localized often in the extracellular space and catalyze the addition of a single ADPR unit, called mono-ADP-ribosylation. Diphtheria toxin–like ADP-ribosyltransferases (ARTDs; also called poly-ADP-ribose polymerases [PARPs]) come in three flavors: (i) without any known enzymatic activity, (ii) as mono-ARTs catalyzing mono-ADP-ribosylation, or (iii) as poly-ARTs, catalyzing the attachment of numerous ADPR moieties, forming poly-ADP-ribose (PAR) chains in the process called poly(ADP-ribosyl)ation (PARylation). ADP-ribosylation is a post-translational modification that serves multiple cellular functions, such as signal transduction, energy metabolism, intracellular trafficking, or cell death (49, 50, 51). Yet, one of its major roles is the maintenance of nuclear homeostasis, regulation of DNA repair mechanism, chromatin remodeling, and transcription (52).

NAD^+^ is also consumed by histone deacetylases known as sirtuins (SIRTs). They use NAD^+^ as an acceptor during the transfer of acetyl (Ac) and other groups from target protein, which is a common post-translational modification, implemented in numerous cellular pathways, including those involved in genomic maintenance (53, 54). Another family of NAD^+^ consuming enzymes are glycohydrolases (NADases), such as CD73, CD38, or sterile α and TIR motif–containing 1. Apart from generating ADPR, this enzyme exhibits ADP-ribosyl cyclase activity, which produces cyclic ADPR, a second messenger in Ca^2+^ signaling. For instance, CD38 is a major extracellular NAD^+^ consumer, and its activity has a wide range of implications in the context of infection, metabolic dysfunction, aging, or tumor biology (55, 56). For more information regarding NAD^+^ multiple cellular roles, we refer the reader to recent reviews (7, 43, 45). In further parts of this review, we focus exclusively on NAD^+^-dependent pathways involved in the maintenance of genome integrity.

## NAD^+^-dependent processes in genome maintenance

There is now a growing body of evidence that NAD^+^-dependent processes are heavily involved in genome maintenance and DNA repair mechanisms (57). This is exemplified in a study by Kiss *et al.* (58), demonstrating that CRISPR–Cas9-generated NMNAT1 U2OS knockout cells show reduced intracellular NAD^+^ levels, which was accompanied by hypersensitivity toward DNA-damaging cisplatin treatment. Furthermore, this study revealed that genetic inactivation of NMNAT1 completely blocked PARP1 activation in the nucleus, leading to increased γ-H2A.X DNA damage foci formation (58). In addition to PARP1, NAD^+^ also fuels the enzymatic activities of other PARPs involved in genome maintenance, such as PARP2, PARP3, TNKS1 (tankyrase 1), and TNKS2 (tankyrase 2). Those PARPs share NAD^+^ as a substrate with the SIRTs, of which some family members, such as SIRT1, SIRT6, and SIRT7, are directly involved in genome maintenance (59). Because of a functional and physical interplay between NAD^+^, PARPs, and SIRTs, the existence of a PARP–NAD–SIRT axis has been proposed that actively supports DNA repair mechanisms (57). In addition, DNA ligases (LIGs) have been suggested as a third class of enzymes that may use NAD^+^ as a substrate to support DNA repair (60). In the following paragraphs, we summarize and discuss the current state of the art on the role of NAD^+^ in functions of these enzymes during genome maintenance.

## ARTDs (aka PARPs)

ARTDs (aka PARPs) are the major consumers of cellular NAD^+^. ARTDs use NAD^+^ to synthesize a monomer or polymer of ADPR (MAR or PAR, respectively) covalently attached to the target molecule. Thereby, ARTDs modify protein residues (Ser, Asp, Glu, Arg, and Lys) or terminal phosphates at DNA breaks (61, 62). During the process, the N-glycosidic bond of NAD^+^ is cleaved and NAM is released. The elongation of PAR chains involves the formation of the glycosidic bond between two riboses: 2′–1′′ for linear chains and 1′–2′′ for branched molecules and can involve up to 200 ADPR units (Fig. 3*A*). Although PARylation is reversible, NAD^+^ is not restored—PAR chains are degraded by poly(ADP-ribose) glycohydrolase to free PAR and/or ADPR molecules because of the cleavage of O-glycosidic bond. The terminal protein-ADPR moiety is cleaved by ADP-ribosyl hydrolases, the macrodomain containing proteins MacroD1/2, or the terminal ADP-ribose protein glycohydrolase 1 (TARG1) (63). The highly dynamic process of PARylation serves multiple cellular roles, including regulation of cellular stress response, survival, energy metabolism, inflammation, host–virus interactions, and many more, as reviewed elsewhere (49, 50). Furthermore, major roles of ARTDs are detection of DNA damage and facilitation of DNA repair and other nuclear mechanism maintaining genomic integrity (52, 64).

The first member identified within the ARTD family, that is, PARP1, accounts for the majority of PARylation activity, both constitutively and upon hyperactivation during DNA damage, when it consumes up to 80% of the nuclear NAD^+^ pool (65, 66, 67). PARP1 acts as a DNA nick sensor, which recognizes, binds to, and becomes activated by DNA strand breaks, and catalyzes immediate PAR synthesis, including autoPARylation (52, 64, 68, 69, 70). That serves as a scaffold for the recruitment of factors involved in different DNA repair mechanism, such as single-strand break repair /BER (*e.g.*, X-ray repair cross-complementing protein 1 [XRCC1]) (71), homologous recombination (*e.g.*, breast cancer type 1 susceptibility protein [BRCA1]), (72) or NHEJ (*e.g.*, DNA-dependent protein kinase catalytic subunit, DNA-PKcs) (73) (more examples can be found in Table 1). PARP1 is recruited to stalled replication forks and helps to stabilize those during DNA damage repair (74, 75). PARP1-mediated PARylation of histone tails results in chromatin relaxation and recruitment of chromatin remodeling proteins, such as SMARCA5 (SWI/SNF related, matrix associated, actin-dependent regulator of chromatin, subfamily A, member 5) facilitating DNA repair (76). Moreover, such chromatin modifications may alter transcription. Further, PARP1 can act as a multitasking transcriptional regulator by binding to promoter sequence of transcription factors (*e.g.*, NF-κB) or to transcription enhancers (*e.g.*, cAMP response element–binding protein) (77, 78). PARP1 activity inhibits the negative elongation factor (NELF), thereby facilitating transcript elongation (79). Other ARTDs family members, particularly PARP2, PARP3, TNKS1, and TNKS2 exhibit partly overlapping as wells as non-redundant functions with PARP1 (Table 1). For a detailed discussion, we refer the reader to previous reviews focusing on the role of ARTDs in DNA damage repair (51, 52, 80, 81, 82, 83), chromatin remodeling (84) and transcription (85, 86). Moreover, PARylation have been also shown to regulate numerous steps in mechanisms maintaining RNA stability, processing, and translation (84, 87, 88, 89), as well as mitotic events and telomere integrity (90).

**Table 1:** NAD^+^-consuming enzymes in the maintenance of genome integrity—major members and targets

Class of enzymes	Specific NAD^+^ consuming members followed by target proteins
ARTDs	PARP1: ATM (226), BRCA1 (72), CHD (227), DDB1–DDB2 (228), DNA-PKcs (73), FUS (229), LIG III (230), MRN (74), NELF (79), RECQ1 (75), SAFB1 (231), SMARCA5 (76), TDP1 (232), Timeless (233), XPA (234), and XRCC1 (71) PARP2: HP1 (235), PARP1, and XRCC1 (236) PARP3: APLF (237), DNA-PKcs, LIG IV, Ku70, Ku80, XRCC4 (238), NuMA, and TNKS1 (239) PARP9: BBAP (240) PARP10: PCNA and RAD51 (241) PARP14: PCNA (242) TNKS1: MDC1 (243) and MERIT40 (244) TNKS2: MDC1 (243)
SIRTs	SIRT1: APE1 (245), Ku70 (246), NBS1 (247), TDG (248), WRN (249), and XPA (250) SIRT2: ATRIP (145), CDK9 (251), and H3 (142) SIRT3: Ku70 (146), OGG1 (148), and RAD52 (147) SIRT6: DNA-PKcs (McCord et al., 2009), H3 (123), Ku80 (Chen et al., 2017), and PARP1 (133, 166) SIRT7: ATM (136), CDK9 (139), H3 (124, 134)
NAD^+^-dependent LIG	LIG IV (60)

Of importance, PARP1 overactivation is believed to determine the fate of the cell following DNA damage (91), which has been linked to patho-mechanisms of numerous disorders and cancer development (92). If DNA damage is low, PARP1 activation contributes to cell survival by mobilizing the DNA repair machinery. But if the damage is extensive, hyperactivation of PARP may cause severe depletion of cellular NAD^+^ levels. ATP-dependent NAD^+^ re-synthesis can lead to an energy crisis and metabolic shifts (*e.g.*, impaired glycolysis, stimulation of pentose phosphate pathway) which cause cellular damage and subsequent cell death *via* necrosis (93). Furthermore, PARP1 regulates p53 activity (94), which initiates caspase-dependent apoptosis, although in the later phase nuclear caspases cleave PARP1, thereby inactivating it and preventing the decline of ATP, which is required for the execution of apoptosis (49, 95). Besides, PAR initiates caspase-independent apoptosis known as parthanatos. Fragments of PAR, released through poly(ADP-ribose) glycohydrolase activity, act as signaling molecules that can be distributed to subcellular compartments and attached to various targets. Binding of PAR fragments to mitochondrial apoptosis-inducing factor (AIF) promotes its nuclear translocation, initiation of large-scale DNA fragmentation, and subsequently cell death (49, 96). Interestingly, knock-in mice expressing the a PARP1 variant (PARP1^D993A^) resulting in hypo-PARylation activity and the formation of hypobranched PAR, revealed compromised genotoxic stress response during replication and led to a shift of the cell fate of mouse embryonic fibroblasts after genotoxic treatment away from DNA repair towards cell death and senescence (97). By these mechanisms, activation of PARP1 after DNA damage, may serve as a molecular switch to determine the post-damage cell fate in terms of DNA repair, cell death, or senescence.

There is room for speculation that besides the quality and quantity of the genotoxic stress, the local and systemic availability of NAD^+^ contributes to such PARylation dependent cell fate decision making. What speaks in favor for such a hypothesis is that PARP1 activity is tightly regulated by the availability and accessibility of NAD^+^. The Michaelis-Menten constant K_m_^(NAD+)^ for major PARPs are similar to or higher than the concentration of free NAD^+^, which indicates that NAD^+^ levels in different subcellular compartments can significantly affect the activity of PARP1 and other PARP family members (98). Under normal conditions, PARP1 has limited NAD^+^ binding ability that accounts for the basal (unstimulated) PARP1 activity, but upon DNA damage, PARP1 interaction with DNA strand breaks leads to allosteric changes that allow full NAD^+^ access to its catalytic site and promotes activation of the enzyme (99). Interestingly, PARP1 activity also stimulates NAD^+^ synthesis, i.e., autoPARylated PARP1 has been shown to bind to NMNAT1, which may provide NAD^+^ to support PARP1 catalytic activity, but also stimulate PARP1 independently of NAD^+^ synthesis (100). Thereby, the interplay between PARP1 and NMNAT1 depends on the phosphorylation state of NMNAT1, which is subject to phosphorylation by protein kinase C (101). Moreover, in response to DNA damage, PARylation has been shown to stimulate the expression of salvage pathway enzymes NAMPT and NMNAT1 (102). Besides, a negative feedback mechanism that prevents excessive depletion of NAD^+^ by PARP1 upon DNA damage has been observed – decreased NAD^+^ levels promoted the binding of PARP1 to DBC1 (deleted in breast cancer 1), a potential tumor suppressor, which inhibits PARP1 activity and leads to DNA damage accumulation (103). Moreover, the NAD^+^–PARP1 interplay fosters crosstalk between subcellular compartments. Nuclear PARP1-dependent NAD^+^ depletion may impact NAD^+^ metabolism in distant regions of the cell (104). On the other hand, mitochondrial (105) and extracellular (21) pools of NAD^+^ have been demonstrated to influence nuclear PARP1 activation and DNA repair. Such observations indicate a complex, multilayer NAD^+^-PARP1 interaction. Taken together, the NAD^+^-dependent process of PARylation has been well established as a critical regulator of DNA damage repair factors, triggering chromatin structural changes, modulating the recruitment of transcription factors, modulating post-transcriptionally the stability of mRNAs, or interfering with mitotic events.

## SIRTs

SIRTs (SITRs) are NAD^+^-dependent deacetylases associated with longevity, aging, and age-related pathologies (106, 107). SIRTs catalyze the removal of an acetyl group from protein lysine (Lys) residues, using NAD^+^ as an acetyl acceptor. During the process cleavage of NAD^+^ N-glycosidic bond releases NAM and the ADPR moiety is covalently attached to the acetyl group on the Lys. Then, the cleavage between acetyl and amino group produces 2-O-acetyl-ADPR (OAADPR) and deacetylated protein (Fig. 3*B*) (8, 108). In mammals, seven homologs are distributed within the cell in the nucleus (SIRT1, 6, 7), cytoplasm (SIRT2), and mitochondria (SIRT3, 4, 5) (109), and the shuttle between cellular compartments has been observed in response to pathological conditions (110, 111). While deacetylation accounts for the main activity, some SIRT family members have been shown to catalyze ADP-ribosylation or removal of different acyl groups, such as malonyl, glutaryl, succinyl, propionyl, myristoyl, and palmitoyl (112, 113). Owing to their wide involvement in post-translational modification, SIRTs are recognized as regulators of diverse biological processes, including energy metabolism (114, 115), inflammation (116), circadian rhythm control (117), redox homeostasis (118), autophagy (119), apoptosis (120), as well as genomic stability (53, 54), and tumorigenesis (108).

Analogously to PARPs, nuclear SIRTs contribute to genome integrity and DNA repair at numerous levels (Table 1). SIRT1 (121, 122), SIRT6 (123), and SIRT7 (124) deacetylate histones leading to the formation of higher-order chromatin compaction, which decreases accessibility for transcription factors. SIRT1 has been shown to directly deacetylate transcription factors, including p53 (125), NF-κB (126), or cAMP response element–binding protein (127), and has been implicated in other aspects of DNA integrity, including replication (128), recombination, DNA damage repair, and tumorigenesis (54, 129). Likewise, SIRT6 (130, 131, 132, 133) and SIRT7 (134) also regulate proteins involved in DNA damage repair. For example, SIRT7 is recruited to DSBs in a PARP1-dependent manner and catalyze H3K122 desuccinylation causing chromatin condensation facilitating damage repair (135). SIRT7-mediated H3 deacetylation affects the accumulation of the DNA damage response factor 53BP1 (134), and direct deacetylation of ataxia–telangiectasia mutated (ATM) kinase promotes activation of various components of DNA damage response (136). SIRT7 has been also implicated in the regulation of ribosome biogenesis and protein synthesis (137) as well as RNA stability (138, 139). SIRT6 association with H3 telomeres has been shown to facilitate the stability of telomeric chromatin (140). Albeit non-nuclear SIRTs have been involved predominantly in the regulation of metabolic homeostasis (107, 141), evidence on their role in genome maintenance is growing. SIRT2 shuttle between cytoplasm and nuclei under pathological and stress conditions has been implicated in the regulation of transcription (142), chromatin condensation during mitosis (111), and DNA damage response (143, 144, 145). SIRT3-mediated deacetylation of Ku70 (146) or RAD52 (147) points to its role in DSBR, whereas modification of 8-oxoguanine-DNA glycosylase 1 (OGG1) facilitates the excision of 7,8-dihydro-8-oxoguanine (8-oxoG) from damaged mitochondrial DNA (148). Overall, the broad and complex involvement of SIRT family members in genomic maintenance is well established and has been a subject on recent reviews (53, 54, 59, 149, 150).

Like PARPs, the activities of SIRTs are tightly regulated by NAD^+^ availability (151, 152), and age-associated decline in NAD^+^ levels and subsequent decrease in SIRT activity have been implicated in the pathogenesis of numerous aging-related diseases (107, 114). Targeting NAD^+^ biosynthesis enzymes such as NAMPT or NMNAT modulate SIRT activity (153, 154). Thus, increased dosage of NAMPT enhanced the transcriptional regulatory activity of the catalytic domain of SIRT2 recruited onto a reporter gene in mouse fibroblasts (153). Furthermore, NMNAT-1 has been shown to interact with SIRT1 in MCF-7 breast cancer cells and was recruited to target gene promoters by SIRT1 to support its deacetylase activity and transcriptional regulation (154). Experiments in mice suggested that modulation of NAD^+^ biosynthesis *via* the *de novo* synthesis pathway and the salvage synthesis pathway activated SIRT1 and prevented ototoxicity of the DNA damaging chemotherapeutic cisplatin (155). In turn, SIRTs affect NAD^+^ biosynthetic pathways to promote an increase in NAD^+^ levels (154, 156), albeit they consume a significantly lower amount of NAD^+^ than PARPs (8). NAMPT was identified as a direct substrate of SIRT6 deacetylation, with a mechanism that upregulates NAMPT enzymatic activity in the cancer cell lines BxPC-3 and MCF-7 (156). Interestingly, SIRT1, but not ARTDs, has been showed to maintain NAD^+^ homeostasis in conditions of excess NAD^+^ availability—SIRT1 silencing prevented intracellular NAD^+^ resetting under prolonged extracellular NAD^+^ exposure in HeLa cells (157).

In addition, a complex interplay between SIRTs and ARTDs occurs, since the enzymes compete for NAD^+^ as a substrate (158, 159). SIRT1 activity seems to be more affected by fluctuating NAD^+^ levels than PARP1 activity, whereas PARP1 displays a higher catalytic turnover of NAD^+^ than SIRT1 (158) that might lead in consequence to reduced SIRT1 activity during PARP1 overactivation following DNA damage (160). SIRT1 (161) and SIRT3 (162) deacetylate PARP1, which blocks its catalytic activity. Moreover, PARP1 and SIRT6 have been shown to bind to each other, which was potentiated by DNA damage (163). Besides crossmodifications, there is evidence on transcriptional coregulation—SIRT1 has been shown to negatively regulate the PARP1 promoter (164), whereas the SIRT1 promoter has been shown to be regulated by PARP2 (165). That adds an additional dimension to SIRT–ARTD crosstalk *via* coregulation of common pathways and targets in maintaining genomic integrity (133, 166).

## NAD^+^-dependent DNA LIGs

In prokaryotes, DNA LIGs can catalyze the ATP/NAD^+^-dependent formation of phosphodiester bonds between adjacent DNA strands, required in the process of replication, recombination, and DNA repair (167). The reaction is initiated by an active site Lys, which attacks the α-phosphate moiety of either ATP or NAD^+^ to form a covalent enzyme–AMP intermediate, and the release of either pyrophosphate (from ATP), or NMN (from NAD^+^). Upon binding to nicked DNA, the AMP moiety is transferred from the Lys to the 5′-phosphate end. Subsequently, the hydroxyl group at 3′ end (3′-OH) attacks the adenylated 5′-phosphate (5′-PO_4_) to establish the new phosphodiester bond between the two ends, which releases AMP and finalizes the ligation (Fig. 3*C*) (168, 169). Mammalian cells have three genes encoding for DNA LIGs—LIGI, LIGIII, and LIGIV, which are essential for normal development and survival (170, 171, 172). Previously, they have been known to use only the ATP for the adenylation, unlike some archaeal LIGs, which utilize either ATP or NAD^+^ (169, 173). Of note, a recent study showed that human LIG IV, involved in the final step of NHEJ, indeed can use NAD^+^ as an alternative substrate for double-stranded DNA ligation (60). Although this finding has been doubted in a subsequent study (174) and requires further confirmation, it questions the exclusiveness of NAD^+^-dependent LIGs in prokaryote and points out a potential caveat in the concept to target them in a novel generation of antibiotics (175).

## NAD^+^-dependent cellular homeostasis

Besides direct reactions mediated by NAD^+^-dependent enzymes, NAD^+^ affects genomic integrity through the maintenance of cellular homeostasis, such as energy production, redox balance, or Ca^2+^ levels. The efficiency of mechanisms involved in genomic stability depends greatly on energy. ATP is required for chromatin expansion, mediated by chromatin remodelers, for example, chromodomain helicase DNA-binding or switch/sucrose nonfermentable (SWI/SNF) (176, 177), which facilitates the recruitment of DNA repair proteins or gene expression. Numerous molecules involved in DNA damage signaling and repair are ATP dependent: DNA LIGs (178), kinases, for example, ATM, ataxia–telangiectasia and Rad3 related (ATR), DNA-PKcs (179, 180), or ATPases such as DNA-binding protein Rad50 (181). Likewise, numerous steps in genome expression and cell division are energy dependent (182, 183).

Disrupted redox balance and accumulation of ROS are major effectors in genotoxic stress. ROS induce DNA damage by oxidation of nucleobases, especially adenine and guanine (8-oxoA and 8-oxo-G), impair the activity of molecules regulating DNA damage response, DNA repair, and other nuclear processes, and influence the activity of transcription factors, modulating gene expression. Such a wide array of ROS-induced alterations affects genomic integrity, contribute to mutagenesis, and cell death (4, 184, 185, 186). An interesting example linking NAD^+^-dependent energy and redox homeostasis with genome maintenance is GAPDH. When oxidized by ROS, it acquires nonglycolytic functions and translocates to the nucleus, where it contributes to the maintenance of DNA integrity, transcriptional regulation, and tRNA export (187).

NAD^+^-derived cyclic ADPR regulates Ca^2+^ signaling (55, 56), which is another important factor in DNA integrity. Dysregulated Ca^2+^ homeostasis leads to mitochondrial accumulation of ROS and subsequently DNA damage (188). Ca^2+^ stimulates proteins involved in DNA damage repair (189, 190), mRNA stability (191), and mitosis (192, 193). Thus, dysregulation of NAD^+^-dependent homeostasis is often deleterious and linked to diseases and cancer (4, 194, 195).

## NAD^+^ supplementation in maintaining genomic stability

Numerous studies confirmed that NAD^+^ status and its supplementation affect genomic stability, DNA repair, and sensitivity to cytotoxic effects of DNA-damaging agents or associated diseases. For instance, Kirkland *et al*. in a number of seminal articles have demonstrated that in rat bone marrow niacin is required for the maintenance of chromosomal stability and facilitates PARP-mediated DNA repair when challenged by the chemotherapy drugs etoposide (12) and ethylnitrosourea (13, 196, 197, 198, 199). These data suggest that niacin supplementation might be beneficial for cancer patients in decreasing the severity of the side effects of chemotherapy (200). NAM supplementation ameliorated the DNA damage induced by exposure of human lymphocytes to various genotoxins, UV irradiation, *N*-methyl-*N*′-nitro-*N*-nitroso guanidine, or dimethyl sulfate, *ex vivo* (201). Our group showed that NA supplementation increased DNA repair efficiency and enhanced genomic stability after X-ray exposure in human peripheral blood mononuclear cells *ex vivo* (10). NR, investigated extensively by Bohr *et al.*, has shown numerous beneficial effects in aging-related and DNA repair–deficient disease models. Xeroderma pigmentosum group A (XPA) is a disorder associated with decreased ability to repair DNA *via* nucleotide excision repair. NR treatment ameliorated numerous endpoints in mitochondrial dysfunction in XPA protein–deficient cells and rescued the life span defect in XPA-1–deficient *Caenorhabditis elegans* (202). Analogously, NR treatment stimulated neuronal DNA repair and improved mitochondrial quality in animal models of ataxia–telangiectasia, a disease linked to ATM deficiency. Moreover, NR supplementation reduced the neuropathological and neurobehavioral disease outcomes and extends the animal life span (203). Cockayne syndrome (CS) is a segmental accelerated aging disorder characterized by progressive neurodegeneration caused by a deficiency of DNA repair proteins CS group A or B. In CS models, because of deficient DNA repair, PARP overactivation leads to decreased cellular NAD^+^ levels and subsequently decreased SIRT1 activity and mitochondrial dysfunction. One-week-long NR treatment reduced CS-associated increased mitochondrial membrane potential or ROS production and normalized the cerebellar transcriptome involved in the mitochondrial pathways, oxidative stress, transcription, DNA repair, DNA damage response, and histone acetylation in CS mice (204). In a mouse model of Alzheimer's disease (3xTgAD mice), which in addition lack the BER enzyme DNA polymerase β, an exacerbation of major Alzheimer's disease features was ameliorated by NR supplementation, such as DNA damage, neuroinflammation, neuronal apoptosis, decreased SIRT3 activity, increased PARP1 activity, but NR also improved cognitive function in multiple behavioral tests and restored hippocampal synaptic plasticity (205). Moreover, short-term NR supplementation prevented the hearing loss in CS mice (206). NR restored mitochondrial quality *via* mitophagy and delayed accelerated aging in *C. elegans* and *Drosophila melanogaster* models of Werner syndrome, a disease caused by mutations in the gene encoding the Werner syndrome (WRN) DNA helicase (207). Furthermore, NR treatment attenuated age-associated and functional changes in hematopoietic stem cells in mice, which was accompanied by significant transcriptional changes of genes involved in DNA damage response and DNA repair (208). Such well and extensively documented benefits of NAD^+^ supplements, particularly NR, imply the universal therapeutic application of these compounds in treating diseases associated with impaired genomic stability. However, because of the complex and still not completely understood cellular function of NAD^+^, the translation to humans remains challenging.

Yet, what has been shown in humans by now is that supplementation with NR ameliorated telomere damage, and several other endpoints of the pathophysiology of dyskeratosis congenita evaluated in primary fibroblasts from dyskeratosis congenita patients (209). Moreover, phase 2 and 3 clinical studies in high-risk patients receiving 500 mg of NAM twice daily for up to 12 months significantly reduced the rates of nonmelanoma skin cancers and actinic keratoses by 10 to 30% compared with placebo control groups. These studies impressively indicate the chemopreventive potential of NAM treatment (210, 211). However, as discussed in the next section, several caveats must be taken into account, when considering the broader use of NAD^+^ boosting molecules as genoprotective agents in humans.

## A double-faced role of NAD^+^ in genome maintenance

With a broad (and constantly growing) number of mechanisms relying on NAD^+^, maintaining its cellular pool emerges as critical to ensure genome integrity and expression. That becomes particularly important in the light of age-related NAD^+^ decline. Therefore, boosting NAD^+^ levels has been considered as a potential therapeutic or preventive factor in multiple pathological conditions (212). In laboratory conditions, supplementation with NAD^+^ precursors, such as NR and NMN, has been shown beneficial against aging and age-related diseases (213, 214). Similar effects have been reached by approaches aiming to increase NAD^+^ levels and metabolism *via* caloric restriction, intermittent fasting, or exercise (7, 106). However, these promising preclinical studies are not easily translated to humans (215). The reason for that might be the complexity of the cellular roles of NAD^+^. For example, the involvement of NAD^+^ in numerous redox reactions may be detrimental for cellular homeostasis when exposed to supraphysiologic NAD^+^ levels (98).

Another issue regarding supplementing NAD^+^ involves the risk of fueling cancer cells. Increased metabolic demands of rapidly growing cancer cells require more NAD^+^ (216). The proliferation-promoting shift toward lactate fermentation in the cytoplasm and reduced oxidative phosphorylation in mitochondria (*i.e.*, the Warburg effect) (217) may impair the NAD^+^/NADH ratio. High NAD^+^ levels may lead to cancer promotion (218). Numerous genes, associated with NAD^+^ biosynthesis and metabolism, have been identified as promoting tumor development, for example, NAMPT (219, 220), nicotinic acid phosphoribosyl transferase (221, 222), NADK (47), which makes them promising targets in cancer therapy. [NB: Yet, NAD^+^ supplementation has also been shown to reduce the incidence and size of tumors (223, 224).] Besides, NAD^+^-consuming enzymes, such as PARPs and SIRTs, play a complex role in cancer development. Under normal conditions, their beneficial role in the maintenance of genome integrity prevents the occurrence of cancer-driving mutations. But at conditions, when tumor already exists, their activity may promote cancer development and resistance to treatment. In such cases, enhancing NAD^+^ levels might have a tumor-promoting effect (14, 225).

## Concluding remarks

Although our understanding of the role of NAD^+^ in nucleic acid maintenance has significantly expanded during recent years, the complexity of NAD^+^ cellular functions makes this topic of relevance in future studies. Many aspects of NAD^+^ metabolism remain unresolved, such as cell type–specific differences or subcellular compartmentalization. Topics, such as the role of NAD^+^ in mitochondrial DNA repair, or NAD^+^ capping, are yet poorly studied and deserve further exploration. Equally important is an understanding and evaluation of the risk–benefit ratio associated with elevating NAD^+^ levels, which may act as a double-edged sword in the context of maintaining DNA integrity and overall NAD^+^-dependent cellular homeostasis. It will be exciting to see how future studies will provide a deeper understanding of these mechanisms.

## Conflict of interest

The authors declare that they have no conflicts of interest with the contents of this article.
